# Waterborne diseases and ethnic-related disparities: A 10 years nationwide mortality and burden of disease analysis from Ecuador

**DOI:** 10.3389/fpubh.2022.1029375

**Published:** 2022-12-21

**Authors:** Esteban Ortiz-Prado, Katherine Simbaña-Rivera, Gabriel Cevallos, Lenin Gómez-Barreno, Domenica Cevallos, Alex Lister, Raul Fernandez-Naranjo, Blanca Ríos-Touma, Jorge Vásconez-González, Juan S. Izquierdo-Condoy

**Affiliations:** ^1^One Health Research Group, Faculty of Health Science, Universidad de Las Américas, Quito, Ecuador; ^2^University Hospital Southampton NHS FT, University of Southampton, Southampton, United Kingdom; ^3^Biodiversity, Environment and Health Research Group (BIOMAS), Faculty of Engineering and Applied Sciences, Universidad de Las Américas, Quito, Ecuador; ^4^Facultad de Ciencias de la Salud, Universidad Latina de Costa Rica, San Pedro, Costa Rica; ^5^Health Management and Research Area, Universidad Internacional Iberoamericana, Arecibo, Puerto Rico

**Keywords:** diseases, water, sanitation, hygiene, disparities, inequalities, WaSH

## Abstract

**Background:**

Despite worldwide progress in terms of clean water supply, sanitation, and hygiene knowledge, some middle and most of low-income countries are still experiencing many diseases transmitted using unsafe water and the lack of sanitation.

**Methods:**

To understand the impact of all waterborne diseases (WBD) registered in Ecuador. We performed a population-based analysis of all cases and deaths due to WBD in Ecuador based on the national public databases of hospital discharges as a proxy of incidence, in-hospital mortality, and countrywide general mortality rates from 2011 to 2020.

**Results:**

In Ecuador, mestizos (mixed European and Indigenous American ancestry) had the greatest morbidity rate (141/100,000), followed by indigenous (63/100,000) and self-determined white patients (21/100,000). However, in terms of mortality, indigenous population have the greatest risk and rates, having a 790% additional mortality rate (2.6/100,000) than the reference group (self-determined white populations) at 0.29/100,000. The burden of disease analysis demonstrated that indigenous had the highest burden of disease caused by WBD with 964 YLL per every 100,000 people while mestizos have 360 YYL per 100,000 and self-determined white Ecuadorians have 109 YYL per 100,000.

**Conclusions:**

In Ecuador, waterborne diseases (WBD) are still a major public health problem. We found that indigenous population had higher probability of getting sick and die due to WBD than the rest of the ethnic groups in Ecuador. We also found that younger children and the elderly are more likely to be admitted to the hospital due to a WBD. These epidemiological trends are probably associated with the lower life expectancy found among Indigenous than among the rest of the ethnic groups, who die at least, 39 years earlier than the self-determined white populations, 28 years earlier than Afro-Ecuadorians and 12 years earlier than the mestizos.

## 1. Introduction

During the last 50 years, the world has experienced a notable increase in wealth in almost every country and region; nevertheless, the rise of inequality has been unparalleled and continuous (1). Several countries' economic prosperity and health development have led to decreased mortality, increased life expectancy, and a continuous struggle to maintain the general state of health (2). However, those on the lower ends of the socioeconomic spectrum have less ability to access optimal welfare conditions such as nutrition, housing, and education. Therefore, they are more likely to suffer from pathogenic infectious diseases caused by micro-organisms such as bacteria, virus or parasites, all responsible for what is known as waterborne diseases (WBD) (1).

WBD are considered an essential indicator of health in the most deprived populations (3). The World Health Organization (WHO) estimates that 844 million people lack a basic drinking water supply services (4). According to UNICEF in 2017, 2.1 billion people in the world did not have access to safe drinking water, which can be contaminated by feces and other wastes, causing diseases such as cholera, dysentery and typhoid fever (5). About 842,000 people die each year from diarrhea due to contaminated water, of which 361,000 are children under 5 years old (6). WHO estimated that 10.6, 15.4, and 45.8 per every 100,000 deaths were attributed to water, sanitation and Hygiene (WaSH) preventable diseases in the Eastern Mediterranean, South East Asia and Africa continents, respectively (7). In Latin America, the consumption of contaminated water and the lack of access to improved sanitation services is related to more than 4,000 premature deaths (8). One important factor is that Latin America is one of the fastest urbanizing regions of the world, where only an average of 30% of the wastewater is treated and most of it is dumped in the natural waterways that are source of water for productive activities and production downstream (9, 10).

Ecuador is one of the most affected countries by this reality, with 15.4% of its urban and 31.8% of the rural population consuming contaminated water with fecal coliforms, and only an average of 20% of wastewater treated (11). In a country like Ecuador, which shares similar characteristics to other countries in the region such as Peru, Colombia, Bolivia or Brazil, a large part of the indigenous population lives in remote areas without adequate access to basic services such as clean-running water, electricity or sewage services (12, 13). It has been reported that Indigenous groups suffer a heavier burden of communicable and non-communicable diseases compared to the rest of the population (14). In Ecuador, only 20.9% of indigenous children have WaSH measures, meaning that 8 out of 10 indigenous children simultaneously lack safe water, basic sanitation or supplies for handwashing (15). The overall low basic sanitation coverage (70.9%) of the indigenous population does not represents the reality of some Amazonian or Andean communities (15). For instance, WaSH within the Amazon region (eastern region of Ecuador), only reaches 24.8%, while in the highlands (central region of Ecuador) and the Coast (western region of Ecuador), this indicator rises to 55.1 and 52.8%, respectively (15).

According to the identifiable Objectives of Sustainable Development of Water, Sanitation and Hygiene of Ecuador, access to safe water is influenced by the ethnicity of Ecuadorians. For instance, only 59.1% of the Afro-Ecuadorian population have access to clean and potable drinkable water, that number raises to 58.7% among Montubios (a group made up of peasant farmers on the Ecuadorian Coast region), 73.4% for mestizos and >95% for self-determined white population (16). In terms of running water, 86.9% of Afro-Ecuadorians, 86.7% of mestizos and 85.6% of Montubios have a constant supply of water for washing their hands and for their lavatories (16).

After 2015, the Sustainable Development Goals (SDG) broadened the spectrum of targets focusing on water, sanitation and hygiene (WaSH), inviting the member states, including Finland in first place with a human development index (HDI) = 0.938, Chile in first place among American countries with HDI = 0.851, followed by Uruguay with HDI = 0.817, while for Ecuador the HDI is 0.759, to fulfill their obligation to bring better services to its population on this matter (17). Unfortunately, the effect of these achievements on health is hard to measure. In Ecuador, two studies found that access to basic services, better living conditions and access to health services reduce morbidity and mortality, both in newborns and in adults (18, 19). Nonetheless, to our best knowledge, there are no studies that explore the national distribution of waterborne diseases and related problems. This information is crucial to guide and to prioritize interventions to address WaSH and WBD related problems among vulnerable populations.

In this context, the aim of this study was to analyze the impact of waterborne diseases on Ecuadorian population and their population differences (age, gender, ethnics, and social health determinants) from 2011 to 2022.

## 2. Methodology

### 2.1. Study design

A country-wide comparison of the total number of cases and deaths attributed to WBD from the 24 provinces and the 221 cantons in Ecuador was performed from 2011 to 2020. We performed a secondary data analysis of publicly available data from the Ecuadorian National Institute of Statistics and Census (INEC) at https://aplicaciones3.ecuadorencifras.gob.ec/sbi-war/.

### 2.2. Setting and participants

Ecuador with an area of more than 283,000 km^2^ is the smallest country in the Andean mountainous region in South America. The country is divided into four geographical regions, the coast, the highlands, the Amazon region, and the Galapagos Islands. The political division encloses 24 provinces, 10 from the highlands, seven from the coast, six from the Amazon region, and one from the insular region of Galapagos. Ecuador has 221 political divisions called cantons and they are comparable to cities elsewhere. In the 2010 Population Census, most of the population self-identified as mestizo (71.9%), followed by those who considered themselves Montubios (7.4%), Afro-Ecuadorians (7.2%), indigenous (7.0%) and white/Caucasians (6.1%). However, mathematical projections as 2021 developed from data of the National Survey on Employment, Unemployment and Underemployment (ENEMDU) 2021 are described in Table 1.

**Table 1:** Population distribution by ethnicity in Ecuador.

**Ethnic self-determination**	**White-Caucasian**	**Afro-Ecuadorian**	**Montubio**	**Indigenous**	**Mestizo**
**Population (** ***N*** **)**	215,499	582,838	744,461	1.401,315	13.458,965
**%**	1.30	3.60	4.50	8.50	82.10
**Urban (** ***N*** **)**	179,079	436,233	320,110	302,036	9.957,615
**Rural (** ***N*** **)**	36,420	146,606	424,351	1.099,278	3.501,349
**Men (** ***N*** **)**	103,748	280,978	376,331	692,399	6.543,623
**Women(** ***N*** **)**	111,751	301,861	368,130	708,916	6.915,342
**Suitable employment (%)**	40.10	29.40	18.60	15.10	35.30
**Unemployment (%)**	7.10	10.20	2.10	1.80	5.60
**Underemployment (%)**	19.90	26.60	31.20	21.20	22.90
**Income poverty rate (%)**	16.90	37.70	38.30	52.70	24.60

### 2.3. Variables

We constructed measurements for disease occurrence using hospital admission rates as a proxy for incidence. In terms of mortality, sex-age and ethnicity specific rates were computed using in-hospital mortality and death certificates as numerators and the population at risk as denominators. The lists of diseases used for the analysis was obtained from the INEC databases (including the variables of year of registration, sex, age, ethnicity, pathology, hospital discharge status, and geographic location) based on The International Statistical Classification of Diseases and Related Health Problems in its 10th version (ICD-10) (Table 2). Due to data source incompleteness determined by the absence of data for the cases studied corresponding to variables such as ethnicity and location especially in the first years reported in this research (2011, 2012, 2013) and underreporting, the proportion of entries with missing data on ethnic identity and educational attainment did not match the cumulative incidence and mortality for sex, age, and place of residence. The data was retrieved as it was documented within the reporting system.

**Table 2:** ICD-10 Classification of waterborne diseases (WBD).

**ICD-10 classification**
A00 Cholera
A01 Typhoid and paratyphoid fevers
A02 Other salmonella infections
A03 Shigellosis
A04 Other bacterial intestinal infections
A05 Other bacterial foodborne intoxications, not elsewhere classified
A06 Amoebiasis
A07 Other protozoal intestinal diseases
A08 Viral and other specified intestinal infections
A09 Other gastroenteritis and colitis of infectious and unspecified origin
A71 Trachoma
B15 Acute hepatitis A
B58 Toxoplasmosis
B68 Taeniasis
B69 Cysticercosis
B75 Trichinosis
B77 Ascariasis
B78 Strongyloidiasis
B79 Trichuriasis
B80 Enterobiasis
B81 Other intestinal helminthiases, not elsewhere classified
B82 Unspecified intestinal parasitism

### 2.4. Data source and measurement

We used the latest estimated projections based on the 2010 National Population Census to compute incidence and mortality sex-age specific rates with a yearly resolution from 2011 to 2020. Information by ethnicity was retrieved directly from the variable “ethnic background” from the official National Institute of Statistics and Census (INEC) databases.

### 2.5. Bias

To reduce the likelihood of incurring in any type of confirmation bias, data was retrieved directly from the official source by two members of the research team. They performed independent analysis and compared the results with the rest of the team. If controversies were observed, the entire team met up to solve any inconsistency.

### 2.6. Statistical analysis

We performed descriptive statistics based on demographic variables to obtain absolute and relative variations. We also constructed measurements for disease occurrence, including in-hospital mortality (%), incidence, and countrywide mortality rates. For the burden of disease analysis, the “lillies” package of the R was used to estimate YLL for patients with a given condition (20), the calculation methodology was based on the number of cases in a population with the diagnosis of a specific disease or condition, using the age of diagnosis of the disease for the cases studied, the Ecuadorian population, and the annual projection for each year based on the life expectancy of the population studied for Ecuador (21).

The analysis of the data was performed using the SPSS statistics software for Mac (IBM Corp. 2014, version 24.0. Armonk, NY, USA) and R version 3.6.2. Figures and graphs were performed in Prism 8 GraphPad Software version 8.2.0 (2365 Northside Dr. Suite San Diego, CA 92108). The basic cartography maps were generated using QGIS Development Team 180 2.8 (Creative Commons Attribution-ShareAlike 3.0 license CC BY-SA).

### 2.7. Ethical considerations

According to the local and international regulation, secondary, fully anonymized publicly available data analysis do no required ethical approval for any kind. All procedures performed in our study were in accordance with the ethical standards of the Minister of Public Health (MoH) and with the Helsinki Declaration and comparable ethical standards.

### 2.8. Availability of data and materials

The datasets generated and/or analyzed during the current study are available in the following link: https://github.com/covid19ec/WASH.

## 3. Results

In the last 10 years of available data, Ecuador officially registered 361,457 cases and 1,870 deaths due to waterborne diseases. Women accounted for 51.8%. The overall incidence rate was 223 cases [CI95% 118–295] per 100,000 people and 1.1 deaths [CI95% 0.7–1.7] (Table 3).

**Table 3:** Number of WaSH related cases and deaths in Ecuador from 2011 to 2020.

	**Cases**	**%**	**Incidence Rate/100,000**	**CI 95%**	**Deaths**	**%**	**Mortality rate/100,000**	**CI 95%**
**Men**	177,704	49.16	221	[115–292]	919	49.14	1.1	[0.8–1.78]
**Women**	183,753	50.84	224	[120–298]	951	50.86	1.1	[0.75–1.75]
**Total**	361,457	100.00	223	[118–295]	1,870	100.00	1.1	[0.78–1.75]

### 3.1. WBD-related morbility

#### 3.1.1. Age and gender differences

The average age of all water borne disease related cases for the mestizo group was 21.2 years. Meanwhile, the indigenous, Afro-Ecuadorian and Montubio groups were younger than mestizos with means of 21.3, 18.6, and 18. One years, respectively. On the other side, the white population recorded a mean of 28.9 years.

In terms of cases by age, we found that 39.9% of all WBD in Ecuador were among children younger than 4 years, followed by those from 5 to 9 years of age with 12.6% of the overall number of cases (Table 4).

**Table 4:** Age differences among patients with Wash related diseases.

**Age**	**Pop. at risk**	**Hospital admissions (*N*)**	**Relative (%)**	**Incidence rate/100,000**	**CI <95%**	**CI > 95%**	**Deaths (*N*)**	**Relative (%)**	**In hospital mortality (%)**
0–4	1,658,115	144,188	39.9	89.18	38.75	124.42	581	31.1	0.4
5–9	1,676,535	45,420	12.6	27.85	16.68	33.43	70	3.7	0.2
10–14	1,682,311	22,429	6.2	13.79	8.18	17.22	37	2.0	0.2
15–19	1,619,198	13,938	3.9	8.62	4.46	12.16	18	1.0	0.1
20–24	1,515,761	14,813	4.1	9.17	4.6	13.16	25	1.3	0.2
25–29	1,397,212	15,554	4.3	9.61	5.18	13.31	23	1.2	0.1
30–34	1,287,159	14,259	3.9	8.81	4.91	12.04	30	1.6	0.2
35–39	1,189,296	11,970	3.3	7.38	4.31	9.65	37	2.0	0.3
40–44	1,076,995	10,354	2.9	6.4	3.48	8.74	31	1.7	0.3
45–49	951,067	9,434	2.6	5.83	3.12	8.22	43	2.3	0.5
50–54	833,293	9,301	2.6	5.74	3.14	7.55	39	2.1	0.4
55–59	719,133	8,703	2.4	5.36	3.13	7.0	48	2.6	0.6
60–64	594,271	8,191	2.3	5.05	2.87	6.53	55	2.9	0.7
65–69	465,854	7,627	2.1	4.7	2.82	5.94	79	4.2	1.0
70–74	347,342	7,091	2.0	4.36	2.71	5.45	90	4.8	1.3
75–79	240,977	6,241	1.7	3.84	2.42	5.01	107	5.7	1.7
80+	256,124	11,944	3.3	7.35	4.54	9.11	557	29.8	4.7
**Total**	17,510,643	361,457	100.0	223.03	118.32	295.33	1,870	100.0	0.5

**Figure 1:**
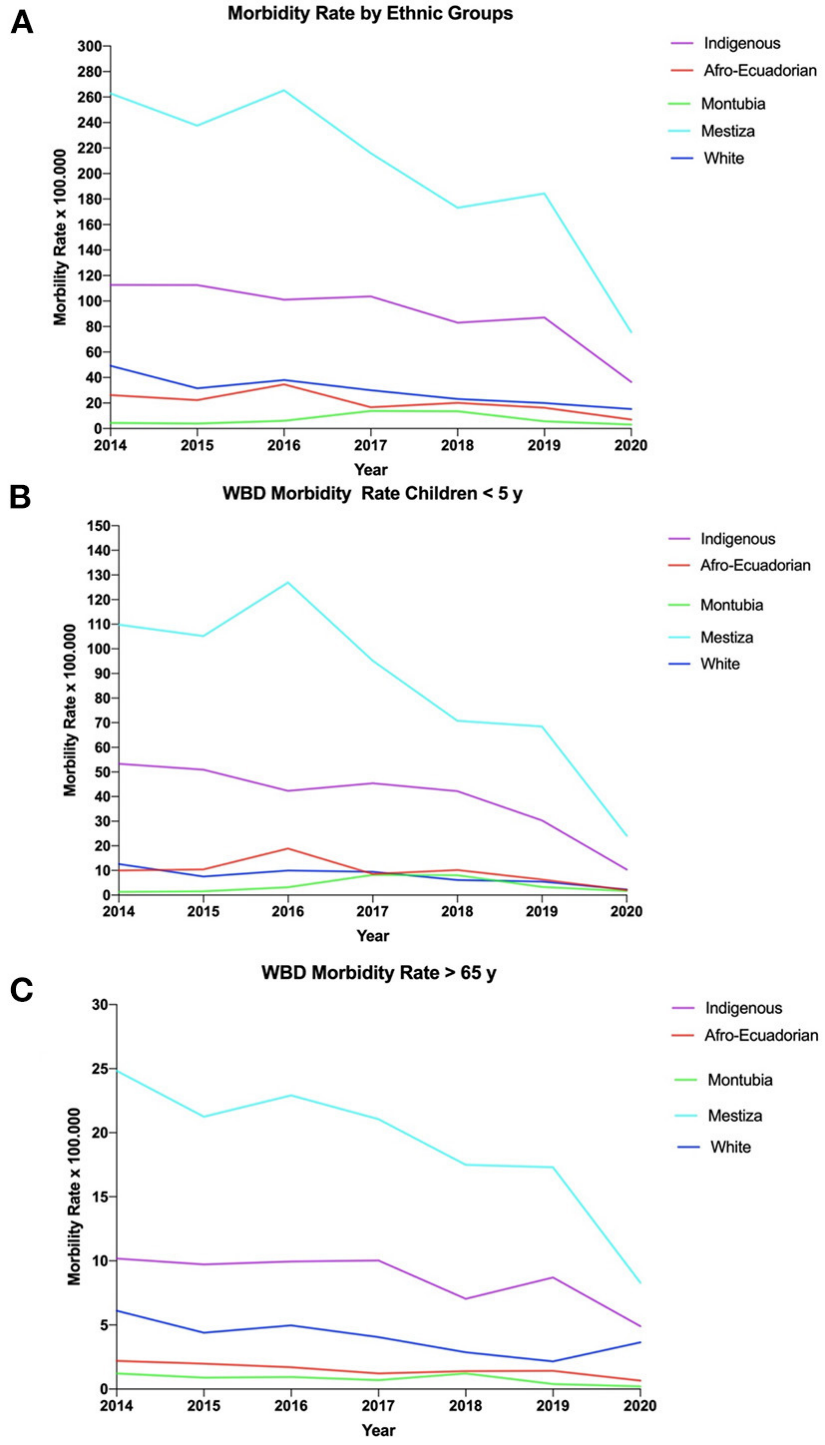
Morbidity due to waterborne diseases in Ecuador from 2011-2020. **(A)** Overall Morbility rate in Ecuador. **(B)** Morbility Rate by ethnic group in children from 0 to 5 years. **(C)** Morbility rate by ethnic group in patients over 65 years of age.

Furthermore, in the subgroup analysis (by age), substantial differences in morbidity among patients under 5 years old per ethnic group were found (Figure 1). In this case, the under 5 years hospital discharge rates showed a 118% increase in hospital discharges rates among mestizos (60/100,000) than indigenous children (27/100,000) and 1,032% more than self-reported white children (5/100,000). In terms of morbidity among those older than 65 years of age, mestizos have a significantly higher incidence rate (13/100,000) than indigenous (6/100,000) and self-reported white elderly (2/100,000) (Figures 1A,B).

#### 3.1.2. Morbidity rates by ethnic groups

The mean overall morbidity rate of WBD in Ecuador was 223 (CI95% 118–295) cases per 10,000 inhabitants (Figure 1). We found that in Ecuador, mestizo people had the greatest morbidity rate (141/100,000) followed by indigenous (63/100,000) and self-determined white patients (21/100,000). However, in terms of mortality, indigenous population have a 790% increase in mortality rate (2.6/100,000) compared to self-determined white populations (0.29/100,000) and 176% more compared to mestizos (0.94/100,000) (Figures 1A,B).

### 3.2. WBD-related mortality

**Figure 2:**
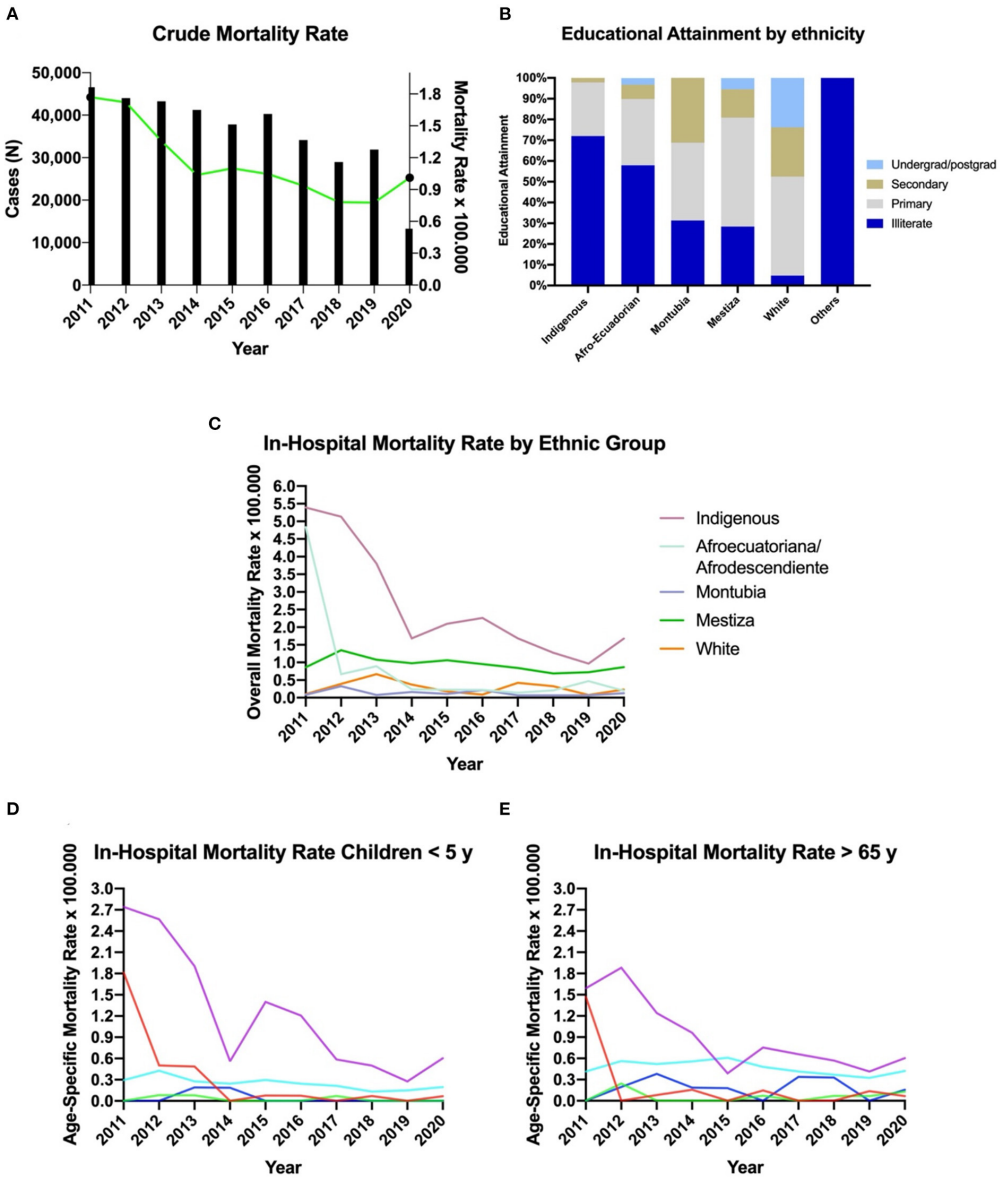
Mortality due to waterborne diseases in Ecuador from 2011 to 2020. **(A)** Mortality rate due to WBD in Ecuador from 2011 to 2020. **(B)** Educational attainment by ethnic group in Ecuador. **(C)** Overall mortality rate by ethnic group. **(D)** Mortality rate by ethnic group in among children younger than 5 years of age. **(E)** Mortality rate by ethnic group in people older than 65 years of age.

In Ecuador, a total of 1,870 deaths were recorded from 2011 to 2020, 951 (51%) were women and 919 (49%) were men. The mean mortality rate of WBD was 1.1 per 100,000 (CI95% 0.7–1.7). From 2011 to 2020, we observed an average decline of 70% in the number of WBD hospital admissions, ranging from 67% from indigenous to 73% among Afro-Ecuadorians (Figure 2A).

#### 3.2.1. Age and gender differences

The average age of death due to WBD in Ecuador as 42.4 for men and 49.6 for women. The difference among ethnic group is significant at 95% confidence level (*p* < 0.001). Most of deaths were found among children younger than 4 years of age, representing 31.07% (*n* = 581) of the total universe, followed by those patients older than 80 years of age, representing 29.79% (*n* = 557).

#### 3.2.2. Mortality rates by ethnic group

In Ecuador, indigenous people have the highest mortality rates due to waterborne diseases per every 10,000 inhabitants. This group has a mortality rate from 2011 to 2020 of 2.6 per 100,000 (CI 95% 1.1–5.2), while Afro-Ecuadorians have a rate of 0.8 per 100,000 (CI95% 0.1–3.0), Mestizos the highest rate of 0.9 per 100,000 (CI95% 0.7–1.2) and self-reported white population with the lowest rate of 0.2 per 100,000 (CI95% 0.08–0.56) (Figure 2C).

Furthermore, in the subgroup analysis per age, substantial differences in mortality among under 5 years old per ethnic group were found (Figure 2D). In this context, the mean mortality rate among indigenous children was 1.2 per 100,000 (CI95% 0.3–2.6), while Afro-Ecuadorians have a rate of 0.3 per 100,000 (CI95% 0.1–1.0), Mestizos with 0.25 per 100,000 (CI95% 0.1–0.3) and self-reported white people with 0.04 per 100,000 (CI95% 0.1-0.19) (Figure 2B). Regarding mortality among the elderly, the trends follow the same pattern than among children (Figure 2C).

#### 3.2.3. Life expectancy

**Figure 3:**
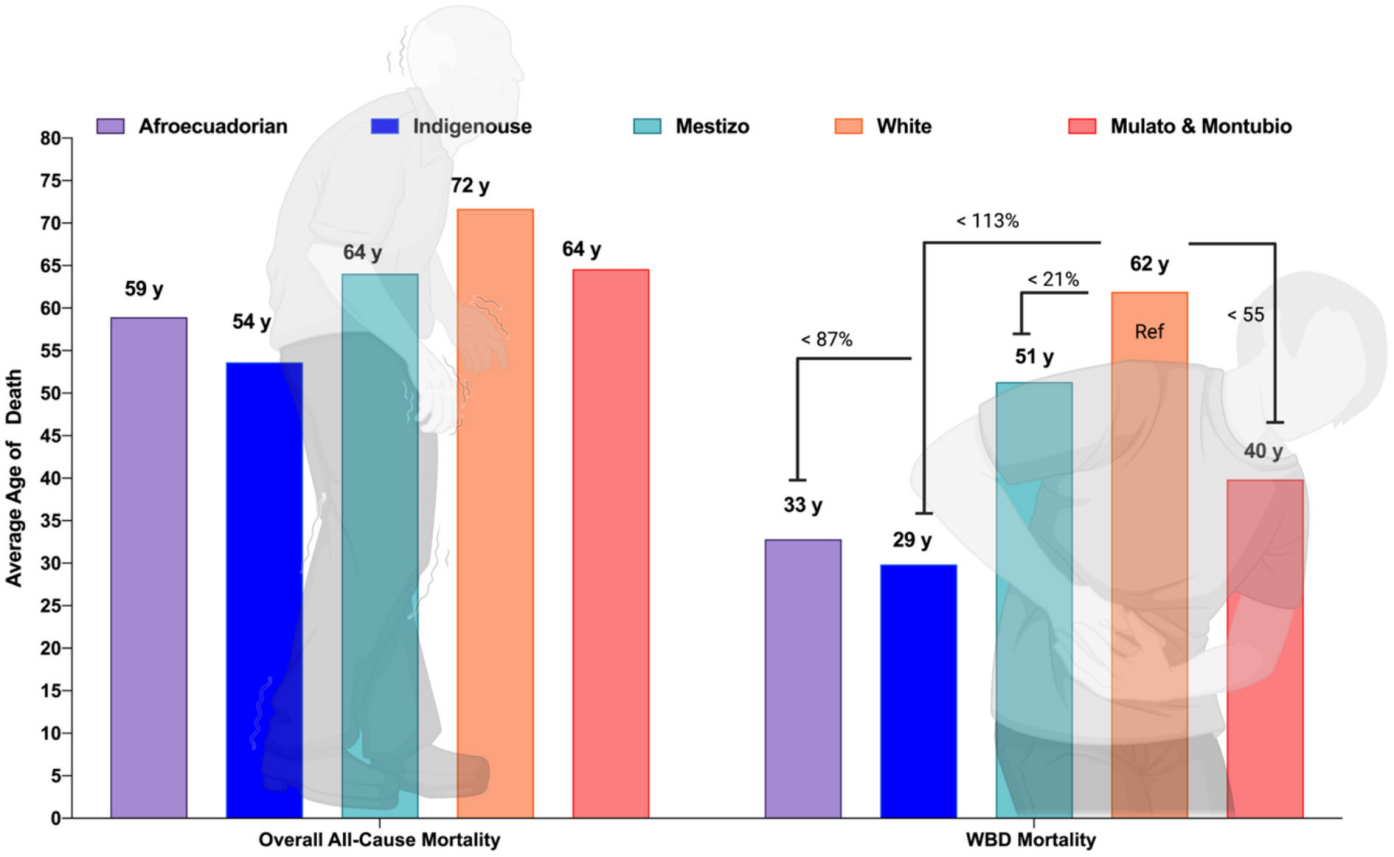
Average age of death due to all cause's mortality and WBD in Ecuador by ethnic group.

In Ecuador, the all-causes mean age at death was 62.3 years while for the WBD the mean age at death was 45.6 years. The average age of death among self-reported white people with a WBD was 63.3 years, while indigenous people died 29.3 years younger than self-reported white people, followed by Afro-Ecuadorians with 28.2 years, Mestizos 12.1 years and Montubios with 5.1 years of age (Figure 3).

#### 3.2.4. In-hospital mortality

Overall, in Ecuador we found an average in-hospital mortality proportion of 5.6% of the total number of admissions. Trichinosis (B75) resulted in the most lethal when admitted to the hospital with 60.0 % of patients dying, followed by Strongyloidiasis (B78) with 12.50 % and other intestinal helminthiases, not elsewhere classified (B81) with 11.76 % (Table 5).

**Table 5:** In hospital mortality (%) caused by WBD in Ecuador from 2011 to 2020.

**Disease**	**ICD-10**	**Deaths (*N*)**	**Hospital admissions (*N*)**	**In hospital mortality (%)**
Typhoid and paratyphoid fevers	A01	13	6,162	0.2
Other salmonella infections	A02	22	11,080	0.2
Shigellosis	A03	1	451	0.2
Other bacterial intestinal infections	A04	229	35,304	0.6%
Other bacterial foodborne intoxications, not elsewhere classified	A05	24	9,259	0.3
Amoebiasis	A06	19	6,413	0.3
Other protozoal intestinal diseases	A07	1	959	0.1
Viral and other specified intestinal infections	A08	9	12,485	0.1
Other gastroenteritis and colitis of inf. and unspecified origin	A09	1,300	262,914	0.5
Acute hepatitis A	B15	46	8,017	0.6
Toxoplasmosis	B58	43	683	6.3
Cysticercosis	B69	123	1,968	6.3
Trichinosis	B75	3	5	60.0
Ascariasis	B77	15	1,282	1.2
Strongyloidiasis	B78	5	40	12.5
Other intestinal helminthiases, not elsewhere classified	B81	2	17	11.8
Unspecified intestinal parasitism	B82	15	4,418	0.3
Total	N/A	1,870	361,457	0.5

### 3.3. Sociodemographic analysis around WBD

#### 3.3.1. Educational attainment

In Ecuador, the number of cases of waterborne diseases, when plotted by educational attainment, demonstrated that from the 100% of deceased people, most of the indigenous have no formal education at all (62%) (Figure 2B). In contrast, self-determined White Ecuadorians with no formal education accounted for <5% in the control group (Figure 2B).

#### 3.3.2. Rural vs. urban differences

The percentage of patients affected by waterborne diseases who lived in urban areas is greater for all groups except for indigenous, where 54% of them are reported to be living in rural areas during the time of diagnosis.

#### 3.3.3. Geographical differences

In terms of provinces, the most affected province in Ecuador was Morona Santiago with 446.23 cases per 100,000 inhabitants, followed by Cañar with 346.47 cases per 100,000 inhabitants and El Oro province with 327.16 cases per 100,000 inhabitants (Table 6).

**Table 6:** Incidence and mortality rate due to waterborne diseases by province in Ecuador from 2011 to 2020.

**Province**	**Hospital admissions**	**Incidence rate/100,000**	**CI < 95%**	**CI>95%**	**Deaths**	**Mortality rate/100,000**	**CI < 95%**	**CI>95%**	**In hospital mortality (%)**
**Azuay**	22,218	275.81	147.85	388.48	164	2.03	0.89	3.09	0.74
**Bolivar**	3,999	198.66	120.08	243.91	20	0.98	0	2.43	0.50
**Canar**	8,943	346.47	204.29	437.83	37	1.45	0.38	3.53	0.41
**Carchi**	2,512	139.65	82.27	183.24	21	1.16	0	2.53	0.84
**Chimborazo**	15,029	300.56	151.26	408.46	116	2.31	1.25	3.37	0.77
**Cotopaxi**	10,689	234.24	125.33	302.75	109	2.46	0.1	6.73	1.02
**El oro**	21,935	327.16	162.77	410.04	52	0.78	0.21	1.57	0.24
**Esmeraldas**	7,190	121.09	59.49	202.07	65	1.09	0.34	2.51	0.90
**Galapagos**	589	202.20	79.29	302.29	0	0	0	0	0.00
**Guayas**	97,223	239.03	122.98	333.74	304	0.74	0.57	0.96	0.31
**Imbabura**	7,408	167.25	86.75	238.23	55	1.23	0.64	1.83	0.74
**Loja**	9,450	191.40	101.43	272.73	67	1.37	0.17	2.8	0.71
**Los rios**	22,656	263.72	119.20	395.87	68	0.79	0.35	1.45	0.30
**Manabi**	30,972	208.25	89.65	311.52	97	0.64	0.35	0.97	0.31
**Morona Santiago**	7,779	446.23	259.82	589.84	37	2.13	0.81	4.3	0.48
**Napo**	3,085	263.33	78.96	465.17	34	2.98	0	10.44	1.10
**Orellana**	2,071	137.85	65.97	215.34	25	1.76	0	8.3	1.21
**Pastaza**	3,030	307.37	134.46	468.22	10	0.95	0	2.82	0.33
**Pichincha**	41,315	140.25	82.81	175.97	341	1.15	0.82	1.56	0.83
**Santa Elena**	11,157	311.18	156.11	397.31	41	1.15	0	2.07	0.37
**Sto. Domingo DLT**	9,474	227.16	148.26	336.32	51	1.2	0.6	1.65	0.54
**Sucumbios**	4,843	237.96	74.90	372.28	43	2.15	0.88	4.7	0.89
**Tungurahua**	15,110	271.86	155.55	378.97	104	1.87	0.77	2.99	0.69
**Zamora C**.	2,780	255.41	177.22	310.17	9	0.84	0	2.44	0.32
Total	361,457	223.55	118.60	296	1,870	1.19	0.83	1.78	0.52

**Figure 4:**
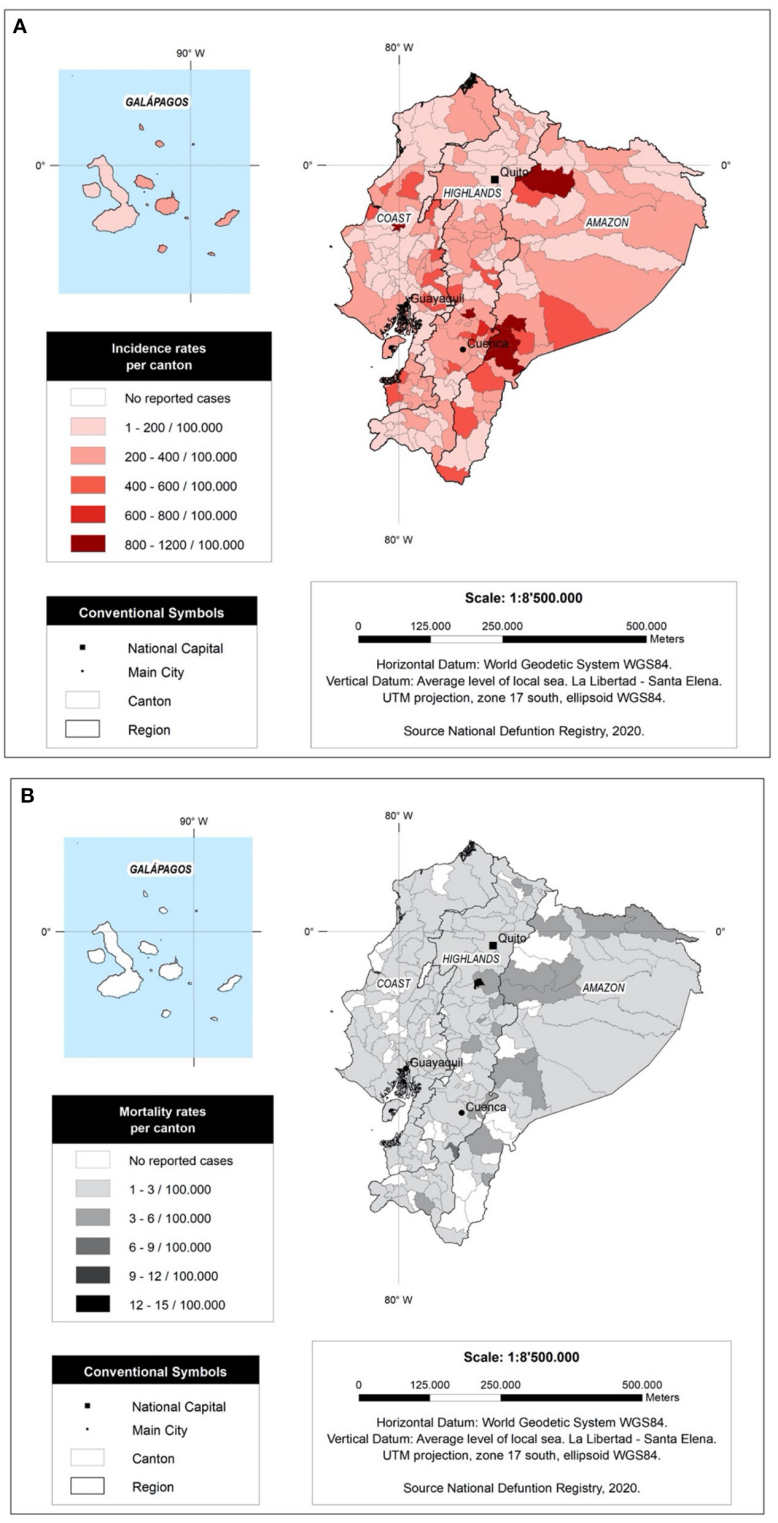
Incidence and mortality rate per 100,000 per canton in Ecuador from 2011 to 2020. **(A)** WaSH related Incidence Rate per canton **(B)** WaSH related mortality rate per canton.

In relation to mortality, Napo Province had the highest mortality rate with at least 2.98 deaths per 100,000 inhabitants, followed by Cotopaxi with 2.46 deaths per 100,000 inhabitants and Chimborazo with 2.31 deaths per 100,000 inhabitants. The most affected cantons (cities) by WBD in Ecuador were Santiago (Morona Santiago) with an incidence rate of 897.33 cases per 100,000 inhabitants, followed by Limon Indanza (Morona Santiago) with 844.98 cases per 100,000 inhabitants and El Chaco (El Napo) with 771.73 cases per 100,000 inhabitants (Figure 4A). In relation to mortality, the canton of Saquisili (Cotopaxi) presented the highest mortality rate with more than 10.59 deaths per 100,000 inhabitants, followed by the canton of Oña (Azuay) with 7.63 deaths per 100,000 cases and Putumayo (Sucumbios) with 4.48 deaths per 100,000 cases (Figure 4B).

### 3.3. WBD burden analysis

Overall, in Ecuador, 56,507 years of life have been lost prematurely due to WBD from 2011 to 2020. Although the majority of the population studied in this analysis was of mestizo ethnicity with 9,822 YLL, the adjusted calculation of YLL per capita (100,000 inhabitants), showed that the indigenous ethnicity group had the highest burden (964/100,000) of disease caused by WBD, followed by mestizos (360/100,000), Afro-Ecuadorians (287/100,000) and self-determined white Caucasians (109/100,000) (Table 7).

**Table 7:** Burden of WBD and years of life lost (YLL) prematurely caused by WBD in Ecuador from 2011 to 2020.

	**White-Caucasian**	**Afro-Ecuadorian**	**Montubio**	**Indigenous**	**Mestizo**	**Other**	**Total**
**Total cases (** ***N*** **)**	23	5	1	847	1,101	27	2,004
**Urban (** ***N*** **)**	718,589	774,486	434,007	218,571	6,904,554	40,579	9,090,786
**Rural (** ***N*** **)**	163,794	267,073	636,721	799,605	3,512,745	12,775	5,392,713
**Total (** ***N*** **)**	882,383	1,041,559	1,070,728	1,018,176	10,417,299	53,354	14,483,499
**YLL**	967	2,992	422	9,822	37,587	4,717	56,507
**YLL/100.000**	**109.6**	**287.3**	**39.5**	**964.7**	**360.8**	**8,840.3**	

Bold values are the total Years of Life Lost Prematurely per 100,000 people.

Consequently, when comparing the indigenous ethnicity with the other ethnic groups, the indigenous were more affected showing an excess of 236.2% of YLL prematurely compared to Afro-Ecuadorians, 2,374.4% compared to Montubios, 167.3% compared to mestizos, and 777.3% compared to White-Caucasians.

## 4. Discussion

**Figure 5:**
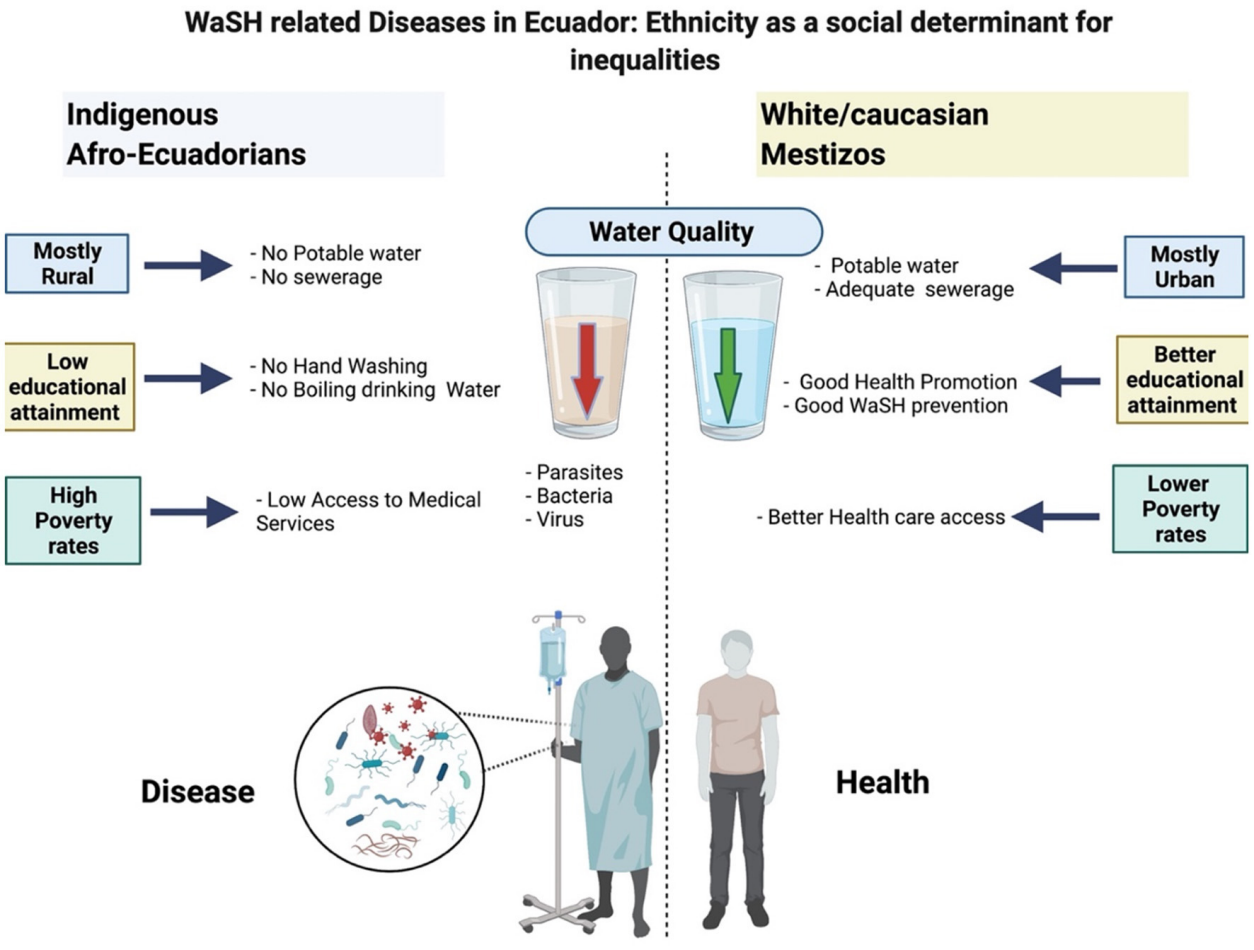
Conceptual framework of the relationship between social determinants of health and water related morbi-mortality.

The results of our research show that in Ecuador there is a high incidence rate of waterborne diseases. In general terms, the burden of disease of these WBD is greater among indigenous populations than among mestizos or self-reported white/Caucasians. This is evident when we analyze the years of life lost prematurely (YYL) due to these infectious diseases related to the consumption of poor-quality water or the lack of sanitation, especially among indigenous populations. We found significant differences between indigenous populations and other ethnic groups in Ecuador, probably linked to higher poverty index rates, lower access to clean water and reduces access to health systems as depicted in our conceptual framework (Figure 5).

According to the local authorities, indigenous people are more likely to live in rural areas, areas with poorer infrastructure, no potable water treatment plants and scarce or none waste management (22, 23). This trend might be related to the fact that rural area has cheaper housing and makes it possible to cultivate the land (24). Therefore, they do not have access to all other favorable social conditions, including education and adequate hygiene habits, since most of them cannot afford housing in urban centers, continuing the vicious circle of poverty among indigenous. Also, these lands suffer high degrees of deforestation, with a consequent loss of water quality that have been linked to gastrointestinal diseases (25, 26).

Apart from that, these populations have low levels of educational attainment which increases the risk of incurring in unhygienic practices such as not washing hands after going to the bathroom or before eating, open defecation and river defecation as well as poor waste management (27, 28). Another finding comes along to the fact that despite having lower WBD incidence rates, indigenous population have higher mortality rates, an indirect indicator of poorer healthcare access increasing the risk of more severe illness, thus, increasing death rates (29).

This study found that the average mortality rate in Ecuador was 1.1 per 10,000 inhabitants, while the hospitalization rate was 223 per 10,000 inhabitants. Countries with a very high Human Development Index, such as Canada, report lower mortality and hospitalization rates from transmitted diseases, on average 0.52 and 12 per 10,000, respectively (30). Regarding nearby realities, in Colombia, the mortality rate for children under 5 years old due to acute diarrheic disease was 2.69 per 10,000 inhabitants during the years 2011–2014. This value did not considered ethnicity as a risk factor, neither age-sex adjusted rates, reducing our chances to fairly compare WBD incidence between countries (31).

It is known that the main factors that contribute to waterborne disease outbreaks are the poor treatment of consumption and waste water, being higher on underground water sources than in other sources (58.6% vs. 53.7%) (32). In Ecuador, 27% of the population consumes polluted water, regardless of the source, being higher in rural and amazon areas where almost 45.3% have inadequate water quality (33). More development in the control of the factors contributing to outbreaks should be considered to achieve the 6th goal in the Sustainable Development Goals (SDGs). Ecuador has made a considerable improvement since the 1990s by increasing some health indicators, such as the percentage of rural population with access to improved water, from 61.4% in 2003 to 75.5% in 2014 (34). In addition, accomplishing the objective of sustainable development in WaSH practices, then water availability seemed to be assured. However, several factors in terms of access and coverage vary, which is the cause of significant disparities. The investment in aqueduct and sewerage systems in Colombia during 2011–2014 were not enough reduce the incidence of waterborne diseases (31). In Argentina and Brazil, although mortality cases decreased, the measure was not enough to eradicate waterborne diseases, neither in the long nor in the medium term (35). In fact, in Colombia and Argentina only 12 and 10 % of the population, respectively, are connected to wastewater treatment facilities (10), which means that wastewaters are dumped in natural waterways without treatment, which can be the source of water for other populations downstream. For example, in Ecuador, a study in Quito, where 98 % of wastewater is dumped directly to waterways, found 26 viral species associated with infections in humans (36).

Indigenous people have limitations to access healthcare, such as geographical isolation and poverty (37). This may be the reason why they show lower rates of hospital discharges but higher rates of mortality due to WBD. Disease progression is another consequence of having poorer access to the health system among indigenous and Afro-Ecuadorian populations. For example, a previous otherwise treatable roundworm infection caused by the ingestion of *Trichinella larvae* becomes severe trichinosis in cases of heavy infestation (38, 39). In addition, several reports show that vital organ complications such as myocarditis, encephalitis, or meningitis, caused by foodborne parasitic diseases increase in-hospital mortality, something that we observed in our results (40, 41).

According to the latest statistics from the National Institute of Statistics, poverty was reduced significantly, especially extreme poverty (42). About WaSH resources, the share of the population using improved drinking water resources in 2015 was 92.6%, and sanitation facilities was 86.1% (34). Despite these achievements, the reduction was not equitable among all ethnic groups in the country. This study shows that WBD in the indigenous population is significantly greater than the one reported among mestizos, Afro-Ecuadorians, and white populations, as shown in another work by Morales and Mideros (43). Living under poverty in rural areas and limited access to clean water, secure food sources and other basic needs have affected this ethnic group. Moreover, there is a strong relationship on land conservation and children's health, especially in rural areas. It has been stated that forest cover is associated with less diarrheal disease in children in developing countries (25). Studies across tropical countries have demonstrate that land conservation is a crucial factor preventing WBD and improving child health. Therefore, land conversion can have tremendous effects on vulnerable population living on rural areas, like indigenous groups in Ecuador.

On the other hand, the diagnosis of less-lethal infectious diseases such as respiratory endocrine and metabolic diseases is more frequent in proportion in the white population, possibly due to better access to health resources and medicines (44). In Ecuador, diseases related to poor hygiene and lack of potable water, clean and safe for health, mainly affect the indigenous population. In a report from the INEC, it was evidenced that the houses with adequate access to clean water did not exceed 80%, while in other groups such as mestizos and white population, it surpassed 97% (42). In general, Ecuadorian indigenous groups have reduced access to safe drinking water and appropriate sanitation and wastewater treatment facilities, which influences greater morbidity and mortality caused by diseases transmitted by the water. All this information is available from the countrywide databases that includes data from indigenous populations that live in Ecuador and have access to health services. In the country however, as well as in other parts of the Amazon rainforest there are indigenous populations living in voluntary isolation that drink water directly from the rivers (45, 46). Those groups could be affected by the poor quality of the water that might arrive through the tributary streams, thus, the importance of WBD is fundamental not only to reduce the prevalence of water-related infectious diseases but also to prevent the potential extermination of indigenous groups living in voluntary isolation (47). The information provided in this investigation seeks to set a precedent and serve as an input to generate public policy related to health prevention and health promotion in the context of WBD.

The effect of WBD on economic and social development was evidenced by a significant number of deaths and a total of 56,507 YLL between 2011 and 2020; while with regard to analyses corresponding to 2016, the disability-adjusted lost years (DALYs/1,000) for low- and middle-income countries in the Americas was much higher (DALY = 799/1,000) compared to countries in the high-income continent (DALY = 25/1,000); However, the regions that were shown to be most affected by diseases related to inadequate water, sanitation and hygiene behaviors were sub-Saharan Africa, and Southeast Asia (48). Although the first analysis exposed the mestizo ethnic group as the most affected by WBDs, the per capita adjusted analysis exposed that the group that truly feels the most WBD burden effects is the indigenous (YLL/100,000 = 964.7), no analyses have been reported that evaluate the effects of ethnic inequities on the disease burden of waterborne diseases, however, we consider that these effects cannot be present only in Ecuador since several developing countries in the world and the region have reported ethnic inequities (49–51).

Finally, we believe that it is essential that both local and national governments focus on providing clean, drinking and readily available water to all the populations that are currently living in regions with not access to it. All the strategies should also aim to achieve adequate management of water sanitation and hygiene with a holistic perspective, including wastewater treatment and land management and to improve other socioeconomic factors within communities.

## 5. Limitations

One of the main limitations we encountered in this study was the quality of the national disease reporting system. As a result, we cannot differentiate the number of people getting sick from the number of events (hospital admissions) that required hospitalization. The same patient could have entered the hospital twice in the same week, but the database cannot differentiate these events. At the same time, we could not purge the database from the presence of garbage codes, which might hinder the accurate analysis of this type of information.

## 6. Conclusion

In Ecuador, waterborne diseases (WBD) are still a major public health problem. We found that indigenous population had higher probability of getting sick and die due to WBD than the rest of the ethnic groups in Ecuador. We also found that younger children and the elderly are more likely to be admitted to the hospital due to a WBD. These epidemiological trends are probably associated with the lower life expectancy found among Indigenous than among the rest of the ethnic groups, who die at least, 39 years earlier than the self-determined white populations, 28 years earlier than Afro-Ecuadorians and 12 years earlier than the mestizos.

## 7. Recommendations

We recommend that all the efforts in terms of public policy need to be directed to improve sanitation and access to clean water based on good data, including the ecological quality of freshwater resources, that provide holistic indicators of water quality and land use (52, 53). In the next 10 years it will be the only chance to overcome disparities and probably make a difference in terms of health outcomes. This is especially relevant in climate change scenarios that can reduce the water availability but also increase the incidence of WBD. Since cultural and social factors are more difficult to change, improving access to basic services such as potable water, waste management and more hygienical environments will represent progress toward equality among groups, preventing infectious diseases with epidemic or pandemic outbreaks such as the one we are currently experiencing with SARS CoV-2 virus, due to, among other causes, the weakness on improved water sanitation systems and good WaSH practices (54).

## Data availability statement

The original contributions presented in the study are included in the article/supplementary material, further inquiries can be directed to the corresponding author/s.

## Ethics statement

Ethical review and approval was not required for the study on human participants in accordance with the local legislation and institutional requirements. Written informed consent for participation was not required for this study in accordance with the national legislation and the institutional requirements.

## Author contributions

EO-P is fully responsible for the conceptualization of the study. EO-P, LG-B, and KS-R contributed to data collection, data extraction data analysis, and data visualization. EO-P, KS-R, LG-B, and GC were responsible for the elaboration of the first draft of the manuscript. DC, JV-G, and JI-C contributed with the discussion section and the final revision of the manuscript. AL and BR-T added important insights from a public health and the water quality-ecological perspective. All authors contributed to the article and approved the submitted version.

## References

[1] OXFAM. Even it up, Time to End Extreme Inequality. Nairobi, Kenya: OXFAM.

[2] Braveman P Gruskin S. Poverty, equity, human rights and health. Bull World Health Organ. (2003) 81:539–45. 12973647 PMC2572503

[3] WHO. Technical Guidance on Water-Related Disease Surveillance. Geneva, Switzerland: World Health Organization. (2011). Available online at: https://www.euro.who.int/__data/assets/pdf_file/0009/149184/e95620.pdf

[4] WHO. Global Water Crisis: Facts, FAQs, and How to Help. Uxbridge, United Kingdom: World Vision (2021). Available online at: https://www.worldvision.org/clean-water-news-stories/global-water-crisis-facts (accessed on August 13, 2021).

[5] UNICEF. 2.1 Billion People Lack Safe Drinking Water at Home, More Than Twice as Many Lack Safe Sanitation. (2017). Available online at: https://www.unicef.org/press-releases/21-billion-people-lack-safe-drinking-water-home-more-twice-many-lack-safe-sanitation (accessed on May 10, 2021).

[6] WHO. Preventing Diarrhoea Through Better Water Sanitation and Hygiene. Geneva, Switzerland: World Health Organization (2014). Available online at: https://www.who.int/water_sanitation_health/diseases-risks/gbd_poor_water/en/

[7] WHO. Water Sanitation & Hygiene for Accelerating and Sustaining Progress on Neglected Tropical Diseases: A Global Strategy 2015-2020. https://www.google.com/search?q=Geneva&stick=H4sIAAAAAAAAAOPgE-LQz9U3sDAxKFcCs4wtDYy0tLKTrfTzi9IT8zKrEksy8_NQOFYZqYkphaWJRSWpRcWLWNncU_NSyxJ3sDLuYmfiYAAAQLfth1MAAAA&sa=X&ved=2ahUKEwiy18r9gdv7AhWHFFkFHQjZCH8QmxMoAXoECHwQAw Geneva, Switzerland: World Health Organization. 10.1093/inthealth/ihv073 PMC5580794 26940305

[8] WHO. Environmental Gradients and Health Inequalities in the Americas., Washington. Geneva, Switzerland: World Health Organization.

[9] Ríos-Touma B Ramírez A. Multiple stressors in the neotropical region: environmental impacts in biodiversity hotspots. In: Multiple stressors in river ecosystems. https://www.google.com/search?q=Amsterdam&stick=H4sIAAAAAAAAAOPgE-LUz9U3MDJLSYpXYgcxs40LtFSzk63084vSE_MyqxJLMvPzUDhWafmleSmpKYtYOR1zi0tSi1ISc3ewMu5iZ-JgAAAXFsvBUQAAAA&sa=X&ved=2ahUKEwjhqb61gtv7AhXjGVkFHWlhA_18QmxMoAXoECHMQAw Amsterdam, Netherlands: Elsevier (2019). p. 205–20. 10.1016/B978-0-12-811713-2.00012-1

[10] Rodriguez DJ Serrano HA Delgado A Nolasco D Saltie G. From Waste to Resource: Shifting Paradigms for Smarter Wastewater Interventions in Latin America and the Caribbean. World Bank, World Bank, Washington, DC. Water Papers.

[11] Larrea CA Greene N. Concentration of Assets and Poverty Reduction in Post-neoliberal Ecuador. In: Dominant Elites in Latin America. New York, NY: Springer (2018). p. 93–118.

[12] Raupp L Cunha GM Fávaro TR. Sanitation conditions of indigenous and nonindigenous households in Brazil according the 2000 and 2010 national censuses. Ciênc Saúde Coletiva. (2020) 25:3753–63. 10.1590/1413-812320202510.04602019 32997009

[13] Hall NL. Challenges of WASH in remote Australian Indigenous communities. J Water Sanit Hyg Dev. (2019) 9:429–37. 10.2166/washdev.2019.154

[14] Romero-Sandoval N Cifuentes L León G. High rates of exposures to waterborne pathogens in indigenous communities in the Amazon region of Ecuador. Am J Trop Med Hyg. (2019) 101:45–50. 10.4269/ajtmh.18-0970 31162016 PMC6609175

[15] INEC. Agua, saneamiento e higiene:Medición de los ODS en Ecuador. Available online at: https://www.ecuadorencifras.gob.ec/documentos/web-inec/Bibliotecas/Libros/AGUA_SANEAMIENTO_e_HIGIENE.pdf (2018).

[16] Diagnostico_ASH_pobreza_INEC_BM.pdf (2016). Available online at: https://www.ecuadorencifras.gob.ec/documentos/web-inec/Bibliotecas/Libros/Diagnostico_ASH_pobreza_INEC_BM.pdf (accessed on October 20, 2021).

[17] United Nations. Water Sanitation – United Nations Sustainable Development. (2015). Available online at: https://www.un.org/sustainabledevelopment/water-and-sanitation/ (accessed on May 10, 2021).

[18] Kuang-Yao Pan W Erlien C Bilsborrow RE. Morbidity and mortality disparities among colonist and indigenous populations in the Ecuadorian Amazon. Soc Sci Med. (2010) 70:401–11. 10.1016/j.socscimed.2009.09.021 19906478 PMC2814897

[19] Weigel MM Sanchez MEC. Ethnic/racial disparities in the fetal growth outcomes of Ecuadorian newborns. J Immigr Minor Health. (2013) 15:198–206. 10.1007/s10903-011-9571-5 22258699

[20] Plana-Ripoll O Canudas-Romo V Weye N. lillies: An R package for the estimation of excess Life Years Lost among patients with a given disease or condition. PLoS ONE. (2020) 15:e0228073. 10.1371/journal.pone.0228073 32142521 PMC7059906

[21] Ortiz-Prado E Espinosa PS Borrero A Cordovez SP Vasconez JE Barreto-Grimales A. Stroke-Related Mortality at Different Altitudes: A 17-Year Nationwide Population-Based Analysis From Ecuador. Front Physiol.; 12(2021). Available online at: https://www.frontiersin.org/articles/10.3389/fphys.2021.733928 (accessed on August 23, 2022). 34675818 10.3389/fphys.2021.733928 PMC8525493

[22] Jiménez A Cortobius M Kjellén M. Water, sanitation and hygiene and indigenous peoples: a review of the literature. Water Int. (2014) 39:277–93. 10.1080/02508060.2014.90345318462504

[23] Brierley CK Suarez N Arora G. Healthcare access and health beliefs of the indigenous peoples in remote Amazonian Peru. Am J Trop Med Hyg. (2014) 90:180. 10.4269/ajtmh.13-0547 24277789 PMC3886418

[24] Gkartzios M Scott M. Residential mobilities and house building in rural Ireland: evidence from three case studies. Sociol Rural. (2010) 50:64–84. 10.1111/j.1467-9523.2009.00502.x

[25] Herrera D Ellis A Fisher B Golden CD Johnson K Mulligan M. Upstream watershed condition predicts rural children's health across 35 developing countries. Nat Commun. (2017) 8:811. 10.1038/s41467-017-00775-2 28993648 PMC5634511

[26] Mosandl R Günter S Stimm B. Ecuador suffers the highest deforestation rate in South America. In: Beck E, Bendix J, Kottke I, et al. (eds) Gradients in a Tropical Mountain Ecosystem of Ecuador. Berlin, Heidelberg: Springer. p. 37–40.

[27] Torres-Slimming PA Wright C Carcamo CP. Achieving the sustainable development goals: A mixed methods study of health-related water, sanitation, and hygiene (WASH) for Indigenous Shawi in the Peruvian Amazon. Int J Environ Res Public Health. (2019) 16:2429. 10.3390/ijerph16132429 31288493 PMC6651388

[28] Halpenny CM Koski KG Valdés VE. Prediction of child health by household density and asset-based indices in impoverished indigenous villages in rural Panamá. Am J Trop Med Hyg. (2012) 86:280. 10.4269/ajtmh.2012.11-0289 22302864 PMC3269282

[29] Ring IT Brown N. Indigenous health: chronically inadequate responses to damning statistics. Med J Aust. (2002) 177:629–32. 10.5694/j.1326-5377.2002.tb04989.x 12463983

[30] Greco SL Drudge C Fernandes R. Estimates of healthcare utilisation and deaths from waterborne pathogen exposure in Ontario, Canada. Epidemiol Infect. (2020) 148:e70. 10.1017/S0950268820000631 32167443 PMC7118719

[31] Rodríguez Miranda JP García-Ubaque CA García-Ubaque JC. Enfermedades transmitidas por el agua y saneamiento básico en Colombia. Rev Salud Pública. (2016) 18:738–45. 10.15446/rsap.v18n5.54869 28453115

[32] Pons W Young I Truong J Jones-Bitton A McEwen S Pintar K. A systematic review of waterborne disease outbreaks associated with small non-community drinking water systems in Canada and the United States. PLoS ONE. (2015) 10:e0141646. 10.1371/journal.pone.0141646 26513152 PMC4625960

[33] INEC. Medición de los indicadores de Agua, Saneamiento e Higiene. (ASH), en Ecuador. (2019). Available online at: https://www.ecuadorencifras.gob.ec/documentos/web-inec/EMPLEO/2019/Indicadores%20ODS%20Agua%2C%20Saneamiento%20e%20Higiene-2019/3.%20Principales%20resultados%20indicadores%20ASH%202019.pdf

[34] The World Bank. Ecuador EC: Health Expenditure: Public: % of Government Expenditure. (2019). Available online at: https://www.ceicdata.com/en/ecuador/health-statistics/ec-health-expenditure-public–of-government-expenditure (accessed on March 2, 2020).

[35] Peranovich A Peranovich A. Enfermedades transmitidas por el agua en Argentina y Brasil a principios del siglo XXI. Saúde E Soc. (2019) 28:297–309. 10.1590/s0104-12902019180378

[36] Guerrero-Latorre L Romero B Bonifaz E. Quito's virome: Metagenomic analysis of viral diversity in urban streams of Ecuador's capital city. Sci Total Environ. (2018) 645:1334–43. 10.1016/j.scitotenv.2018.07.213 30248857

[37] United Nations. State of the World's Indigenous Peoples. (2015). USA: United Nations. Available online at: https://www.un.org/development/desa/indigenouspeoples/wp-content/uploads/sites/19/2018/03/The-State-of-The-Worlds-Indigenous-Peoples-WEB.pdf.

[38] Berger S Informatics G. Trichinosis: Global Status 2010 Edition: Global Status 2010 Edition. https://www.google.com/search?q=Sebastopol&stick=H4sIAAAAAAAAAOPgE-LUz9U3MEwrskxS4gAx85LN07S0spOt9POL0hPzMqsSSzLz81A4Vhmpi_SmFpYlFJalFxYtYuYJTkxKLS_IL8nN2sDLuYmfiYAAAmta-BFgAAAA&sa=X&ved=2ahUKEwidu8iQhNv7AhWLElkFHW_LAakQmxMoAHoECGkQAg Sebastopol, CA: O'Reilly Media, Inc. (2010).

[39] Ortega-Pierres MG Arriaga C Yépez-Mulia L. Epidemiology of trichinellosis in Mexico, Central and South America. Vet Parasitol. (2000) 93:201–25. 10.1016/S0304-4017(00)00342-3 11099838

[40] Chávez-Ruvalcaba F Chávez-Ruvalcaba MI Santibañez KM. Foodborne Parasitic Diseases in the Neotropics–a review. Helminthologia. (2021) 58:119–33. 10.2478/helm-2021-0022 34248373 PMC8256457

[41] Tanabe MB Schilling M White AC. Neurocysticercosis and Other CNS Helminthic Infections. In: Neurological Complications of Infectious Diseases. Berlin, Heidelberg: Springer (2021). p. 225–254.

[42] INEC. Reporte de pobreza por consumo en Ecuador 2006-2014. (2015). Available online at: http://www.ecuadorencifras.gob.ec/documentos/web-inec/Bibliotecas/Libros/reportePobreza.pdf

[43] Morales M Mideros A. Análisis de la pobreza multidimensional en los hogares de la agricultura familiar campesina en el Ecuador, 2009-2019. Rev Econ. (2021) 73:7–21. 10.29166/economa.v73i118.3379

[44] Ortiz-Prado E Ponce J Cornejo-Leon F. Analysis of Health and Drug Access Associated with the Purchasing Power of the Ecuadorian Population. Glob J Health Sci. (2016) 9:p201. 10.5539/gjhs.v9n1p201

[45] Amorim FF. Povos indígenas isolados no Brasil e a política indigenista desenvolvida para efetivação de seus direitos: avanços, caminhos e ameaças1. Rev Linguística Antropológica. (2016) 8:19–39. 10.26512/rbla.v8i2.16298

[46] Bodley JH. Isolated tribes: Human rights first. Science. (2015) 349:798–9. 10.1126/science.349.6250.798-b 26293944

[47] Ortiz-Prado E Cevallos-Sierra G Vasconez E. Avoiding extinction: the importance of protecting isolated Indigenous tribes. Altern Int J Indig Peoples. (2021) 17:130–5. 10.1177/1177180121995567

[48] Prüss-Ustün A Wolf J Bartram J. Burden of disease from inadequate water, sanitation and hygiene for selected adverse health outcomes: An updated analysis with a focus on low- and middle-income countries. Int J Hyg Environ Health. (2019) 222:765–77. 10.1016/j.ijheh.2019.05.004 31088724 PMC6593152

[49] Viáfara-López CA Palacios-Quejada G Banguera-Obregón A. Inequidad por la condición étnico-racial en el aseguramiento de salud en Colombia: un estudio de corte transversal. Rev Panam Salud Pública. (2021) 45:e18. 10.26633/RPSP.2021.18 33500690 PMC7820510

[50] Valdivia M. Sobre los determinantes étnico-culturales de la inequidad en salud materno-infantil en el Perú. In: Salud, interculturalidad y comportamientos de riesgo. Lima: GRADE Group for the Analysis of Development (2011). p. 85–118.

[51] Bucheli M Scuro L United Nations Development Programme (eds). Población afrodescendiente y desigualdades étnico-raciales en Uruguay. [Montevideo], Uruguay: PNUD (2008).

[52] Ríos-Touma B Acosta R Prat N. The Andean Biotic Index (ABI): revised tolerance to pollution values for macroinvertebrate families and index performance evaluation. Rev Biol Trop. (2014) 62:249–73. 10.15517/rbt.v62i0.15791 25189082

[53] Jaureguiberry P Titeux N Wiemers M Bowler DE Coscieme L. The direct drivers of recent global anthropogenic biodiversity loss. Sci Adv. (2022) 8:eabm9982. 10.1126/sciadv.abm9982 36351024 PMC9645725

[54] Ortiz-Prado E Simbaña-Rivera K Gómez-Barreno L Guerra CA. Clinical, molecular and epidemiological characterization of the SARS-CoV2 virus and the Coronavirus disease 2019 (COVID-19), a comprehensive literature review. Diagn Microbiol Infect Dis. (2020) 98:115094. 10.1016/j.diagmicrobio.2020.115094 32623267 PMC7260568

